# Case Report: Pembrolizumab unmasks a capillary leak syndrome with chylothorax in early TNBC

**DOI:** 10.3389/fonc.2026.1786343

**Published:** 2026-05-22

**Authors:** Naïla Benkalfate, Ghazi Hadjamara, Caroline Petorin, Magaly Zappa, Aimable Habonimana, Dufens Pierre Louis, Anne-Laure Chene, Jean-Patrick Clarke, Renan Liberge, Kinane Drak Alsibai, Claire Defrance, Barbara Pistilli, Baptiste Boulet, Soraya Benguerfi, Houari Aissaoui

**Affiliations:** 1 Department of Pulmonology, Cayenne University Hospital, French Guiana, France; 2 Department of Pathology, Cayenne University Hospital, French Guiana, France; 3 Department of Oncology, Cayenne University Hospital, French Guiana, France; 4 Department of Radiology, Cayenne University Hospital, French Guiana, France; 5 Department of Internal Medicine, Cayenne University Hospital, French Guiana, France; 6 Department of Pulmonology, Institut du Thorax, Nantes Université, Centre Hospitalo-Universitaire (CHU) Nantes, Nantes, France; 7 Pulmonology Outpatient Clinic, ELSAN Santé Atlantique Clinic, Saint-Herblain, France; 8 Department of Medical Imaging, Nantes Université, CHU Nantes, Nantes, France; 9 Gustave Roussy Cancer Center, Villejuif, France; 10 Medical Intensive Care Unit, Nantes University Hospital, Nantes, France

**Keywords:** capillary leak syndrome (CLS), chylothorax, immune-related adverse event (irAE), pembrolizumab, triple-negative breast cancer (TNBC)

## Abstract

Pembrolizumab has become standard of care in early-stage triple-negative breast cancer (TNBC), but its expanding use may induce rare adverse events involving vascular and lymphatic endothelium. Capillary leak syndrome (CLS) remains an exceptionally rare toxicity of PD-1 blockade, scarcely reported in breast cancer, especially in the adjuvant setting. We report the case of a 40-year-old female with early-stage TNBC who presented with progressive dyspnea, diffuse subcutaneous edema and bilateral pleural and peritoneal effusions after two cycles of adjuvant pembrolizumab. Imaging confirmed polyserositis with interstitial pulmonary edema, and laboratory tests showed hypoalbuminemia without other abnormalities. Thoracentesis revealed milky pleural fluid containing chylomicrons, confirming chylothorax. Bronchoalveolar lavage, medical pleuroscopy, and pleural biopsies demonstrated a normal-appearing pleura without malignant or inflammatory infiltrates. Extensive workup ruled out infectious, neoplastic, cardiac, renal, and autoimmune causes, supporting pembrolizumab-induced vascular and lymphatic endothelial dysfunction. Corticosteroid therapy (1 mg/kg) led to rapid clinical and radiologic resolution. Pembrolizumab was permanently discontinued with no recurrence after corticosteroid tapering. This case highlights a rare but clinically significant immune-related adverse event (irAE) characterized by both vascular and lymphatic capillary leak under pembrolizumab. With PD-1 inhibitors increasingly used in curative TNBC, unexplained serous effusions and edema should prompt consideration of CLS and warrant comprehensive diagnostic evaluation to enable timely immunosuppression.

## Introduction

Pembrolizumab, an anti-programmed cell death 1 (anti-PD-1) monoclonal antibody, has demonstrated significant improvement in survival in early-stage triple-negative breast cancer, as shown in the phase 3 KEYNOTE-522 clinical trial combining pembrolizumab with chemotherapy in both neoadjuvant and adjuvant settings (1).

While its use improves outcomes, immune checkpoint inhibitors (ICIs) can cause irAE, affecting multiple organ systems.

Capillary leak syndrome (CLS), first described in 1964 by Bayard D. Clarkson (2) in its idiopathic form, may also occur secondary to immunotherapy. It is a rare but potentially life-threatening immune-related adverse event (irAE), characterized by plasma leakage from the vascular compartment into the interstitial space, leading to hypoalbuminemia, hemoconcentration, and fluid accumulation (e.g., edema, pleural or peritoneal effusions) (3), sometimes associated with chylothorax, reflecting both vascular and lymphatic endothelial injury.

We report a case of pembrolizumab-induced CLS with chylothorax in an early-stage TNBC patient, supported by an extensive diagnostic evaluation and successfully treated with corticosteroids. This case contributes additional insight into the spectrum of immune-mediated endothelial toxicities associated with PD-1 blockade.

## Case presentation

A 40-year-old woman with no significant past medical history, non-smoker, was diagnosed in June 2024 with TNBC of the right breast. Initial imaging revealed a 28×36 mm lesion in the upper outer quadrant with bilateral axillary lymphadenopathy. Biopsy confirmed grade III invasive ductal carcinoma (ER-/PR-/HER2-, Ki-67 80%). Genetic testing was negative for BRCA and other predisposition mutations.

She received neoadjuvant chemotherapy with carboplatin-paclitaxel-pembrolizumab in September 2024 followed by epirubicin-cyclophosphamide-pembrolizumab from October to December 2024. Imaging showed partial response and no immune-related adverse event occurred during this neoadjuvant therapy. Breast-conserving surgery with sentinel node biopsy performed in January 2025 showed complete pathological response with post-treatment fibrosis. Adjuvant radiotherapy was performed in April 2025, and pembrolizumab was resumed on 18 April 2025 (adjuvant setting; planned 9 cycles); she received 2 infusions (cycle 1 on 18 April 2025 and cycle 2 in early May 2025).

**Figure 1:**
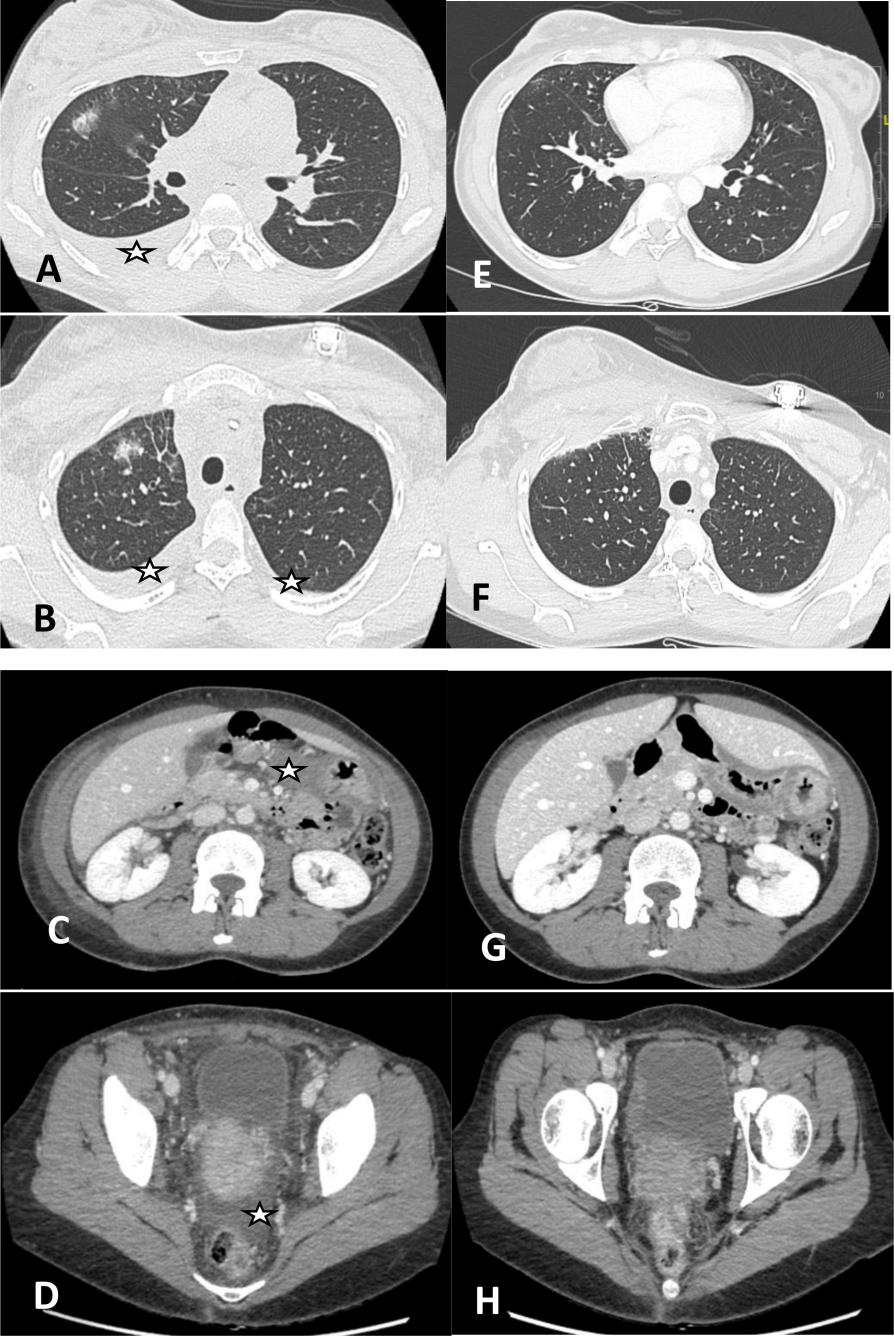
Chest and abdominal CT scans before and after corticosteroid therapy. **(A, B)** Bilateral pleural effusions, interlobular septal thickening, and areas of ground-glass opacity are evident prior to treatment. **(C, D)** Small-volume peritoneal effusion is also visible. **(E–H)** Follow-up scans one week after corticosteroid taper show complete resolution of pleural and peritoneal effusions and normalization of pulmonary parenchyma.

Thirty days after the first pembrolizumab infusion and one week after the second one, she developed progressive dyspnea, cervicofacial and diffuse subcutaneous abdominal edema resulting in weight gain but with normal blood pressure. Echocardiography was normal except a small pericardial effusion; NT-proBNP was negative, no diuretics were administered. The thoracic-abdominopelvic CT scan revealed bilateral pleural effusion, interlobular septal thickening in the right upper lobe with patchy infiltrates, minimal ascites, and diffuse subcutaneous edema (**Figure 1**). Laboratory findings included hypoalbuminemia (31.6 g/L), normal renal/hepatic function, no proteinuria, CRP 5 mg/L, normal TSH, and CA15–3 of 9.1 U/mL; hematocrit remained within normal range, with no evidence of hemoconcentration. VEGF levels were normal. Autoimmune workup revealed low-titer ANA (1:320), anti-SSA positivity, and weakly positive direct Coombs test without clinical features of connective tissue disease. Minor salivary gland biopsy was normal (Chisholm score 0). The patient had no sicca or extra-respiratory manifestations and was evaluated by an internal medicine specialist, who favored pembrolizumab-induced autoimmunity in this context.

**Figure 2:**
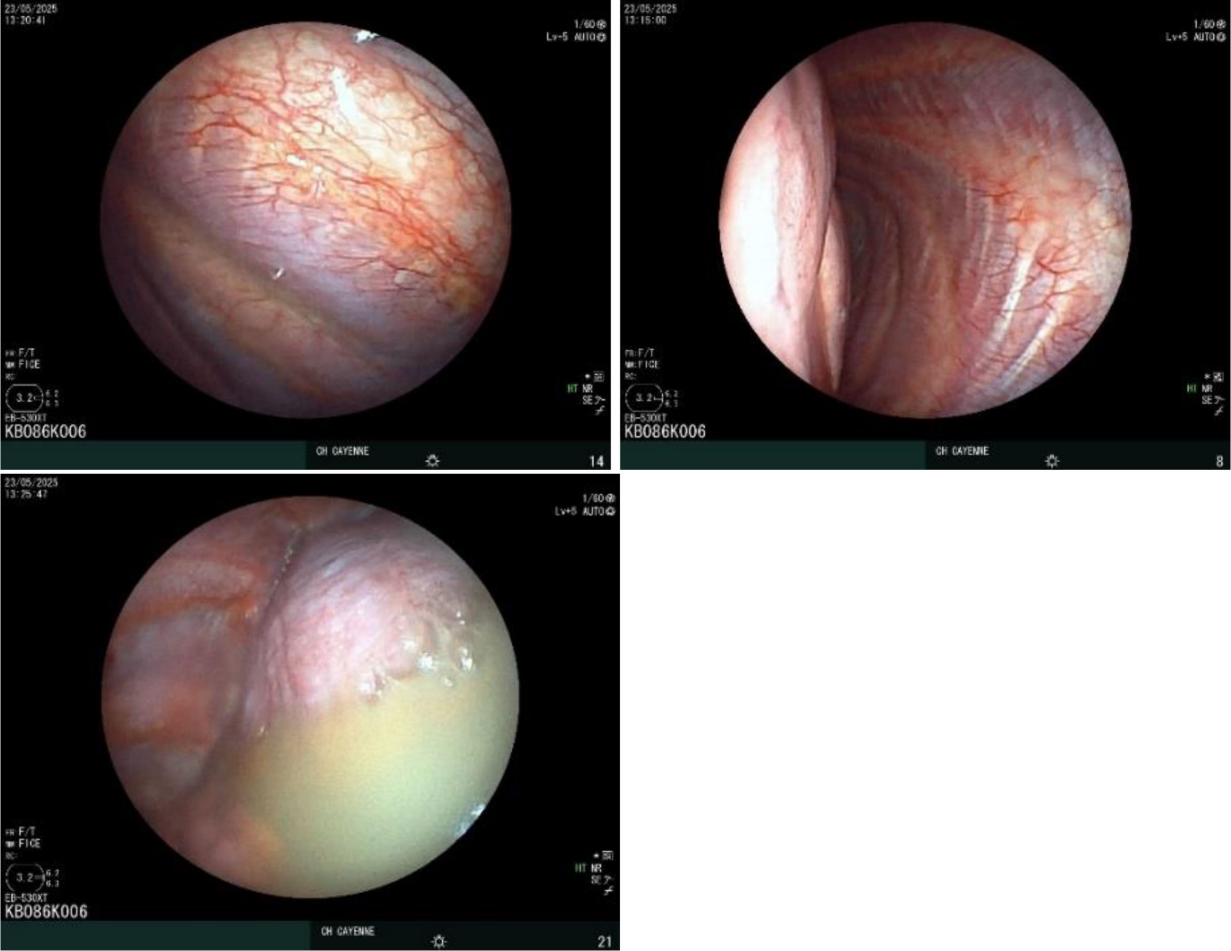
Medical pleuroscopy demonstrating normal-appearing parietal and visceral pleura without evidence of inflammation or malignancy. Milky chylous effusion is present, consistent with chylothorax.

Thoracentesis yielded an exudative effusion. Pleural fluid characteristics are summarized in **Table 1**. Thoracentesis yielded an exudative effusion with a milky appearance and confirmed chylomicrons, consistent with chylothorax. Serum triglyceride and cholesterol levels were not available due to local technical limitations; however, the diagnosis was considered robust given the characteristic appearance and biochemical findings. Cultures for bacteria, mycobacteria, fungi (including Histoplasma), and parasites were negative. Cytology revealed foamy macrophages, neutrophils, rare lymphocytes, and intact mesothelial cells without atypia or malignant cells. Pleural adenosine deaminase was low. Medical pleuroscopy showed a macroscopically unremarkable pleural cavity, and biopsies revealed no inflammatory infiltrate or malignancy (**Figure 2**). Bronchoalveolar lavage was unremarkable.

**Table 1:** Pleural fluid analysis.

Parameters	Pleural fluid
Appearance	Milky
Chylomicron	Present in large amount*
Triglycerides (mmol/L)	0.83 (0.73 g/L)
Total protein (g/L) // protein ratio (pleural/serum)	42 // 0.7
Total LDH (IU/L) // LDH ratio (pleural/serum)	66 // 0.6
Cytology	Mixed pleural cellular infiltrate without predominance
Microbiology**	Negative

*Lipoprotein electrophoresis of the pleural fluid: chylomicrons 12.7%, consistent with a high level. **This included an extensive microbiological workup covering parasitology, mycology, bacteriology, and mycobacteriology.

Given the absence of infectious, malignant, cardiac, renal, or autoimmune causes, and the recent exposure to pembrolizumab, a diagnosis of pembrolizumab-induced CLS with associated lymphatic capillary dysfunction was established.

The patient underwent three therapeutic thoracenteses (600–1000 mL each), followed by initiation of oral corticosteroids on 12 June 2025 (1 mg/kg/day for 2 weeks, then tapered over 4 weeks with a weekly 10 mg reduction). This led to rapid and sustained resolution of symptoms by 19 June 2025 and radiologic abnormalities; follow-up CT in August 2025 after steroid discontinuation confirmed complete resolution (**Figure 1**). She did not require albumin infusion or dietary modifications. A multidisciplinary tumor board recommended permanent discontinuation of pembrolizumab.

**Figure 3:**
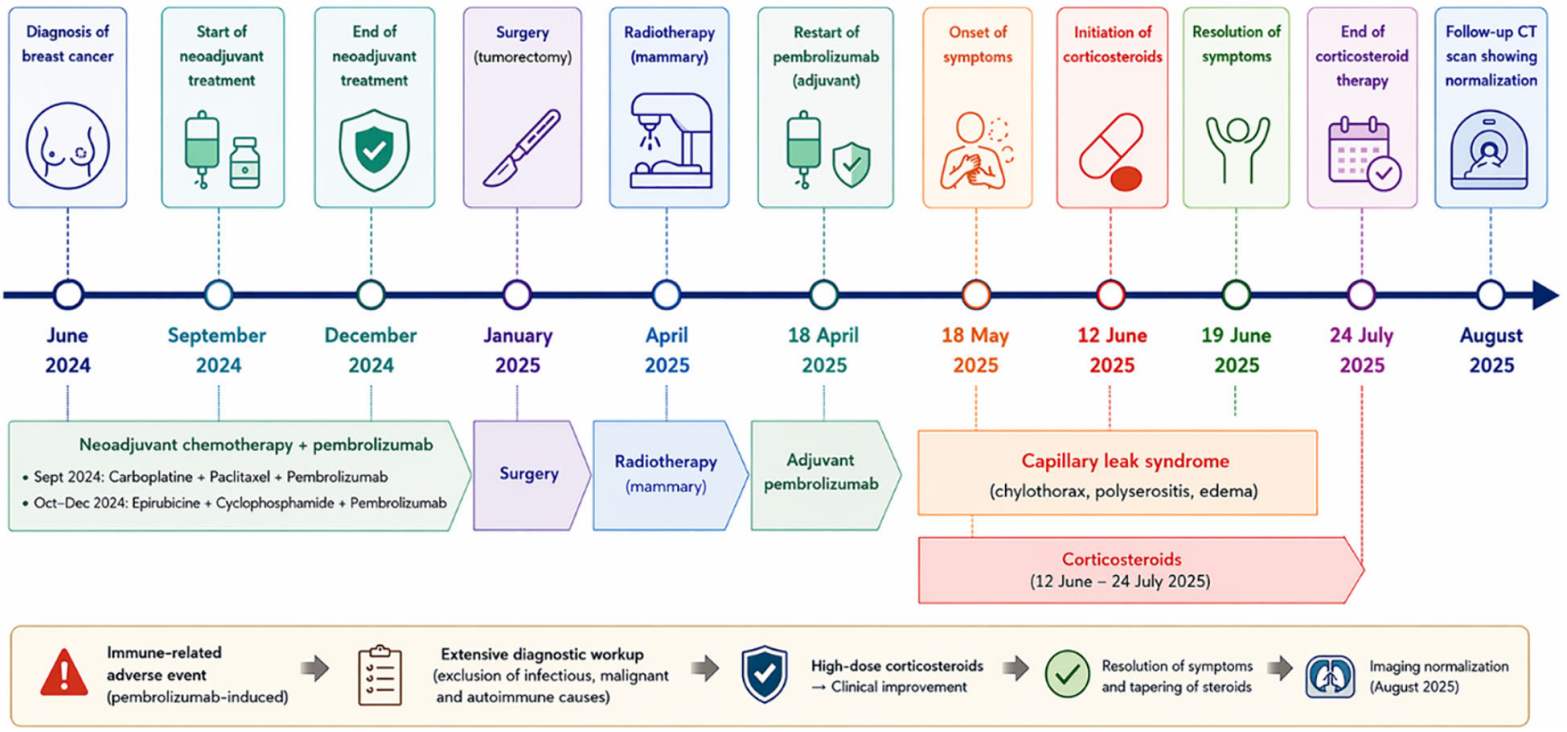
Patient's clinical timeline to key events.

A timeline summarizing key clinical events has been included in accordance with CARE guidelines (**Figure 3**).

## Discussion

Immune checkpoint inhibitors (ICIs) have revolutionized cancer therapy, but can lead to a spectrum of irAEs, including rare and potentially life-threatening complications such as CLS. CLS is typically characterized by hypoalbuminemia, edema, and serous effusions, sometimes without hypotension or hemoconcentration, complicating the diagnosis (3).

The association of systemic CLS and lymphatic capillary dysfunction under pembrolizumab has been described in other reports (4). These events likely represent a continuum of immune-mediated endothelial activation and barrier dysfunction. CLS occupies the “leak phenotype” end of this spectrum, characterized primarily by increased capillary permeability rather than structural destruction. In this case, the absence of pleural inflammatory infiltrates on biopsies supports a functional, immune-mediated disruption of endothelial junctions rather than true pleuritis, reinforcing the concept of non-inflammatory endothelial toxicity under PD-1 blockade.

Immune-mediated endothelial dysfunction may be triggered by cytokines such as IL-2, IL-6, TNF-α, and VEGF (4). PD-1 inhibition amplifies cytotoxic CD8+ T-cell activity, which may inadvertently target endothelial cells, promoting permeability. Chylothorax suggests that lymphatic endothelial structures were similarly affected.

This case stands out for several reasons. First, it illustrates an early-onset, atypical CLS phenotype with polyserositis and chylothorax in a patient receiving adjuvant pembrolizumab, highlighting that irAE may develop even after prior neoadjuvant tolerance. A systematic review and global pharmacovigilance study by Wong So et al. showed that ICI-associated CLS tends to present with a less abrupt onset (median time to symptoms 12 weeks) and less hemoconcentration compared to the idiopathic form of CLS, consistent with our patient (5).

Importantly, no standardized diagnostic criteria exist for ICI-associated CLS, and diagnosis relies on adapted features from classical CLS combined with a temporal association with ICI exposure and the exclusion of alternative etiologies. While classical idiopathic CLS is typically defined by the triad of hypotension, hemoconcentration, and hypoalbuminemia (3), ICI-related cases frequently present with an incomplete phenotype, often lacking hypotension and hemoconcentration (5). In our patient, the presence of hypoalbuminemia, diffuse edema, pleural and peritoneal effusions, and a clear temporal relationship with pembrolizumab, together with a comprehensive negative etiological workup, strongly supported the diagnosis of ICI-associated CLS despite the absence of hemodynamic compromise. To further contextualize the diagnosis, **Table 2** compares the defining features of idiopathic systemic capillary leak syndrome (Clarkson disease) and ICI-associated CLS, mapped against the clinical findings observed in our patient (**Table 2**).

**Table 2:** Comparison of classical capillary leak syndrome (CLS), immune checkpoint inhibitor (ICI)-associated CLS, and findings in our patient (3–5, 9).

Feature	Idiopathic SCLS (clarkson disease)	ICI-associated capillary leak syndrome	Our patient’s features
Epidemiology	Ultra-rare (~500 cases reported); mean age ~51 y; no sex predominance	Very rare (~0.19% of ICI-treated patients for immune-related generalized edema); median age ~62 y	40 year-old woman
Monoclonal gammopathy	Present in 82–97% of adult cases	Not a typical feature	Absent
Onset pattern	Abrupt, prodrome 6–12 h (flu-like symptoms, myalgias, weakness)	More insidious; median onset ~12 weeks after ICI initiation (IQR 8–49 weeks)	30 days after resuming pembrolizumab, but exposed 5 months before onset of symptoms
Clinical course	Recurrent, relapsing-remitting episodes with asymptomatic intervals	Usually a single episode; recurrence uncommon if ICI discontinued	Single episode without relapse after pembrolizumab discontinuation
Triad	Hypotension + severe hemoconcentration + hypoalbuminemia	edema/serositis + hypoalbuminemia; Hypotension and hemoconcentration less consistent	Edema/serositis; minor hypoalbuminemia 31.6 g/L, no hemoconcentration
Preceding irAE	N/A	57% had another irAE before CLS onset	No other irAE before
Complications	Compartment syndrome, rhabdomyolysis, VTE, flash pulmonary edema, multi-organ failure	Chylothorax, serositis, organ dysfunction	Chylothorax, no organ dysfunction
Acute management	Symptomatic (vasopressors, colloids)	Corticosteroids (82.5% of cases); high-dose prednisone ≥1 mg/kg in 55%	Corticosteroids 1 mg/kg during 2 weeks, tapered over 4 weeks (10 mg/week)
Prophylaxis/Long-term	Monthly IV immunoglobulins (IVIg); theophylline + terbutaline between episodes	ICI discontinuation (permanent in ~35%); IVIg reported effective in refractory cases	ICI discontinuation

The incomplete phenotype—including chylothorax without hypotension—demonstrates that CLS presentations can be subtle but clinically meaningful. Reported cases in the literature vary widely in presentation, from isolated peripheral edema to severe anasarca or chylothorax (6–8), underscoring the diagnostic challenge and the importance of considering CLS in the differential diagnosis of unexplained fluid overload in ICI-treated patients.

Furthermore, the extensive diagnostic workup—including pleuroscopy with biopsy, pleural fluid analysis, bronchoalveolar lavage, imaging, and autoimmune and infectious testing—allowed exclusion of alternative etiologies and strengthened the diagnosis of pembrolizumab-induced CLS.

Prompt corticosteroid therapy (1 mg/kg/day) resulted in rapid and sustained resolution without adjunctive immunosuppression. In contrast, refractory cases in the literature have required tocilizumab, anakinra, anti-VEGF therapy, intravenous immunoglobulins, or plasma exchange (6, 7, 9). Rechallenge with ICIs is generally discouraged due to recurrence risk.

Epidemiologically, the REISAMIC study, a French prospective registry of ICI-related adverse events, provides important context. Among more than 1,000 patients treated with ICIs between 2014 and 2020, only 20 cases of ICI-related generalized edema or CLS were reported (prevalence 0.19%), primarily in melanoma and lung cancer, with no cases in breast cancer. Seventy-five percent of patients received corticosteroids, resulting in partial or complete responses in two-thirds, but mortality reached 20%, highlighting the potential severity of this rare irAE even when managed appropriately (9). Our case underscores that early recognition and timely corticosteroid intervention can prevent progression and achieve rapid clinical resolution.

With pembrolizumab now a standard component of curative-intent therapy in early-stage TNBC, awareness of rare endothelial irAE is increasingly critical. This case highlights a reversible but potentially serious toxicity with implications for surveillance and management. Importantly, it demonstrates that lymphatic and vascular endothelial injury may occur simultaneously under PD-1 blockade, providing mechanistic insight into how ICIs disrupt microvascular homeostasis. The stepwise diagnostic approach presented here—integrating pleural fluid biochemistry, endoscopic evaluation, histopathology, and exclusion of competing causes—offers a model for clinicians confronted with unexplained serous effusions in patients receiving ICIs.

Regarding ICI types, most CLS cases in the literature are associated with anti–PD-1 (nivolumab, pembrolizumab) or anti-cytotoxic T-lymphocyte-associated protein-4 (anti–CTLA-4) (ipilimumab) agents, used either alone or in combination (9). Our case adds to the limited number of CLS reports in patients with early-stage breast cancer treated with pembrolizumab, broadening the spectrum of tumors and clinical contexts where CLS may occur. Other immunotherapies and targeted agents have also been implicated such as anti-CD20 monoclonal antibodies, CAR T-cell therapies and tyrosine kinase inhibitors. While the incidence remains low, the study emphasizes that CLS is a potential class effect across multiple immuno-oncologic agents, requiring awareness beyond checkpoint inhibitors alone (10).

## Conclusion

Given the expanding use of immune checkpoint inhibitors (ICIs) in early-stage cancer treatment, rare irAE such as CLS and lymphatic capillary dysfunction warrant heightened clinical vigilance. This case expands the spectrum of pembrolizumab-associated endothelial toxicities and underscores the importance of prompt recognition, thorough diagnostic evaluation, and timely corticosteroid therapy. Further study is needed to elucidate the mechanisms by which PD-1 inhibition perturbs vascular and lymphatic barrier integrity.

## Data Availability

The datasets presented in this article are not readily available because not applicable: case report. Requests to access the datasets should be directed to naila.benkalfate@chu-nantes.fr.
